# Gemcitabine with or without continuous infusion 5-FU in advanced pancreatic cancer: a randomised phase II trial of the Italian oncology group for clinical research (GOIRC)

**DOI:** 10.1038/sj.bjc.6602640

**Published:** 2005-06-28

**Authors:** F Di Costanzo, P Carlini, L Doni, B Massidda, R Mattioli, A Iop, E Barletta, L Moscetti, F Recchia, P Tralongo, S Gasperoni

**Affiliations:** 1 U.O di Oncologia Medica, Azienda Ospedaliera Careggi, Via Pieraccini 17, Florence, Italy; 2 U.O di Oncologia Medica A, Polo Oncologico S Raffaele Regina Elena, Roma, Italy; 3 Oncologia Medica, Policlinico Universitario, Cagliari, Italy; 4 U.O di Oncologia Medica, Ospedale S Croce, Fano, Italy; 5 Servizio di Oncologia, Ospedale di Latisana, Udine, Italy; 6 Oncologia B, Istituto Nazionale Tumori Pascale, Napoli, Italy; 7 Oncologia Medica, policlinico Umberto I, Roma, Italy; 8 U.O di Oncologia Medica, Ospedale di Avezzano, Italy; 9 U.O di oncologia Medica, Ospedale G Di Maria, Avola, Siracusa, Italy

**Keywords:** 5-FU, gemcitabine, advanced pancreatic cancer

## Abstract

This study was performed to determine the activity of adding continuous infusion (CI) of 5-fluorouracil (5-FU) to gemcitabine (GEM) *vs* GEM alone in advanced pancreatic cancer (APC). In all, 94 chemo-naïve patients with APC were randomised to receive GEM alone (arm A: 1000 mg m^−2^ per week for 7 weeks followed by a 2 week rest period, then weekly for 3 consecutive weeks out of every 4 weeks) or in combination with CI 5-FU (arm B: CI 5-FU 200 mg m^−2^ day^−1^ for 6 weeks followed by a 2 week rest period, then for 3 weeks every 4 weeks). Overall response rate (RR) was the primary end point and criteria for decision were planned according to the Simon's optimal two-stage design. The overall RR was 8% (arm A) and 11% (arm B) (95% confidence interval: 0.5–16% and 2–22%), respectively, and stable disease was 29 and 28%. The median duration of RR was 34 weeks (range 25–101 weeks) for GEM and 26 weeks (range 16–46 weeks) for the combination. The median progression-free survival (PFS) was 14 weeks (range 2–65 weeks) and 18 weeks (range 4–51 weeks), respectively. The median overall survival (OS) was 31 weeks (range 1–101 weeks) and 30 weeks (1–101 weeks). Toxicity was mild in both arms. This study does not show promising activity in terms of RR, PFS and OS for the double combination arm in APC.

Pancreatic cancer is the fifth most common cancer worldwide. Unfortunately, most patients have locally advanced or metastatic pancreatic cancer and usually are poorly responsive to chemotherapy (Greenlee *et al*, 2001). Randomised studies have revealed a significant better overall survival (OS) with chemotherapy against best supportive care (BSC) (Weinerman and MacCormick, 1994; Glimelius *et al*, 1996). Although the increase in median survival was modest: 4 and 6 months for the treatment group as compared with 3 and 2.5 months for BSC, respectively, various schedules and different regimens have been evaluated with no proven benefit over single-agent 5-fluorouracil (5-FU) (Cullinan *et al*, 1985; Cullinan *et al*, 1990). In advanced pancreatic cancer (APC), continuous infusion (CI) of 5-FU is superior to 5-FU bolus in terms of response rate (RR) and toxicity (Hansen *et al*, 1988; Hidalgo *et al*, 1999).

Gemcitabine (GEM), a deoxycitidine analogue, has demonstrated activity in APC in terms of clinical benefit and survival (Rothenberg *et al*, 1996). A phase III trial compared GEM *vs* 5-FU alone, the GEM arm showed a statistically longer survival and better clinical benefit (Burris *et al*, 1997). Phase I and II trials of GEM plus 5-FU bolus reported interesting results in terms of clinical benefit (38–66%). Recently, Eastern Cooperative Oncology Group (ECOG) randomised patients with APC to 5-FU plus GEM *vs* GEM alone. The OS was better, but not significant in the combination arm (6.7 *vs* 5.4 months; *P*=0.09) and grade 3 and 4 leukopenia, thrombocytopenia and diarrhoea was higher in the combination arm. Several combination regimens with 5-FU, cisplatin, capecitabine, irinotecan, oxaliplatin, premetrexed and docetaxel were developed without a sure advantage (Cartwright *et al*, 2002; Kindler, 2002; Lutz *et al*, 2002; Schilsky *et al*, 2002; Pizzolato and Saltz, 2003; Schneider *et al*, 2003).

The current multicentre randomised phase II study was started to investigate the feasibility and activity of the combination with GEM plus CI 5-FU *vs* GEM alone in patients with APC. The rationale of the combination of 5-FU with CI and GEM is to prolong thymidylate synthetase inhibition, increasing the chance of pharmacodynamic with GEM and to reduce toxicity with respect to 5-FU bolus.

## MATERIALS AND METHODS

### Patient eligibility

The following inclusion criteria were mandatory to the study: histological or cytological diagnosis of APC (locally advanced or metastatic) with bidimensionally measurable disease. Patients were not amenable to surgery or radiotherapy. Karnofsky performance status (KPS) was 50 or greater, age between 18 and 75 years, no prior chemotherapy, no central nervous system metastases, life expectancy of at least 3 months, adequate haematological (neutrophil count >1500 dl^−1^; platelet count >100 000 dl^−1^), renal (serum creatinine <1.5 × the upper limit of normal (ULN) value) and hepatic (alkaline phosphatase <3 × ULN value and bilirubin <1.5 × ULN value) functions. The protocol was approved by the Ethical Committee and written informed consent was obtained from all patients.

### Treatment plan

Patients were centrally randomised by the central office of the Italian Oncology Group for Clinical Research (GOIRC) to receive: GEM alone (*arm A*) or in combination with CI 5-FU (*arm B*). GEM was administered in both arms as a 30-min intravenous (i.v.) infusion at the dose of 1000 mg m^−2^ once per week for 7 weeks, followed by 2 weeks of rest. Thereafter, GEM was administered once weekly for 3 consecutive weeks out of every 4 weeks. In arm B, GEM was combined with CI 5-FU 200 mg m^−2^ day^−1^ i.v. for 6 weeks in the first cycle, followed by a week of rest and then for 3 weeks every 4 weeks. All patients were treated on outpatient basis.

### Pretreatment evaluation and assessment of efficacy

At screening, all patients were evaluated with physical examination, complete blood count with leucocyte differential count, serum creatinine, bilirubin, transaminase, and alkaline phosphatase, serum tumour marker (CEA and Ca 19-9) levels, ECG and computed tomography (CT) scan of chest, abdomen and pelvis of all tumour sites. During the study, patients were monitored before each cycle for medical history, physical evaluation, clinical benefit, serum chemistry and tumour markers, and evaluated for toxicity. The RR (WHO) were assessed every 2 months or earlier if clinically indicated. If a response was documented, treatment continued until progression. Clinical evaluation of response was not used. Pleural effusion, ascites and hepatomegaly were not used for response assessment. In the case of grade 2 nonhaematological toxicity or greater, patients received symptomatic therapy and 5-FU therapy was stopped until the toxicity ceased and the dose was reduced in the next cycle as follows: in the case of grade 2 stomatitis, diarrhoea or HFS, dose reduction was about 25% of planned dose; in the case of grade 3 stomatitis, diarrhoea or HFS, dose reduction of 5-FU was 50%; and in the case of grade 4 stomatitis, diarrhoea or HFS, 5-FU and GEM therapy was discontinued. In the case of grade 2 haematological toxicity, GEM dose was reduced by 25%; in the case of grade 3, dose reduction was 50%; patientsdiscontinued from therapy when treatment was associated with grade 4 neutropenia and infection. Patients before each cycle received a diary for evaluation of quality of life (QoL), according to Subjective Chemotherapy Impact (SCI) questionnaire (Tamburini *et al*, 1991). The questionnaire allowed us to assess the duration of physical and psychological discomfort of patients during the treatment. The questionnaire consisted of two items: (1) how many disturbed days did you have during the treatment?; (2) how many days do you want to cancel during the treatment? The questionnaire was answered before each cycle.

### Statistical considerations

Primary end points of this study were RR. Secondary objectives were to assess the OS, safety and tolerability of the regimens. Overall survival was calculated from the first day of treatment until the date of death. Patients alive at the end of study were censored at last observation visit. For progression-free survival (PFS), the last date PFS was the date of progression or death (if dead without progression). Patients not experiencing progression or death were censored at last observation visit. Progression-free survival and OS were described according to the Kaplan–Meier method. Descriptive statistics formed the primary basis of analysis for the data collected in this study. The study was not powered for a formal comparison of efficacy outcome measures, and these were not performed. For each arm, a Simon's optimal two-stage design was chosen. According to this approach, the expected sample size is minimised for the first stage if the regimen has low activity. It was considered that the minimum RR had to be at least 10% and combination would had been investigated further if it showed an RR of 25% or more. Alpha and beta errors were fixed at 5 and 20%, respectively. According to these assumptions, 18 patients per arm had to be recruited in the first step if at least two responses were observed, then a further 25 patients would have been enrolled, up to a total of 43 patients per arm. A minimum of eight responses was defined in order to claim activity.

## RESULTS

### Patient demographics

After 30 months of accrual, 94 patients were enrolled into this trial and their characteristics are listed in Table 1. Three patients were ineligible due to nonpathologic diagnosis of APC (*n*=1), synchronous second primary tumour (*n*=1) and nonmeasurable disease: pleural effusion (*n*=1). The two arms of treatment were well balanced for prognostic factors. The median age was 63 years (range: 34–75 years). The KPS was ⩾80 in 62 patients (68%) and <80 in 29 patients (32%). In the GEM arm, 35 patients (73%) had metastatic disease and 29 patients (67%) in the combination. In all, 48 patients (53%) had primary pancreatic tumour plus metastases in the liver.

### Toxicity

A total of 253 cycles of chemotherapy were administered and the median number of cycles was 2 (range: 1–6). In all, 90 patients in both arms were evaluable for toxicity. The data toxicity of four patients were missing. The most common side effects are shown in Table 2. Both chemotherapy regimens were well tolerated. The principal differences between the two regimens were grade 1 and 2 mucositis (arm A/B: 14 *vs* 29%) and diarrhoea (4 *vs* 20%). One patient in the combination arm had grade 4 thrombocytopenia. There were no treatment-related deaths.

### Tumour response, PFS and survival

**Figure 1:**
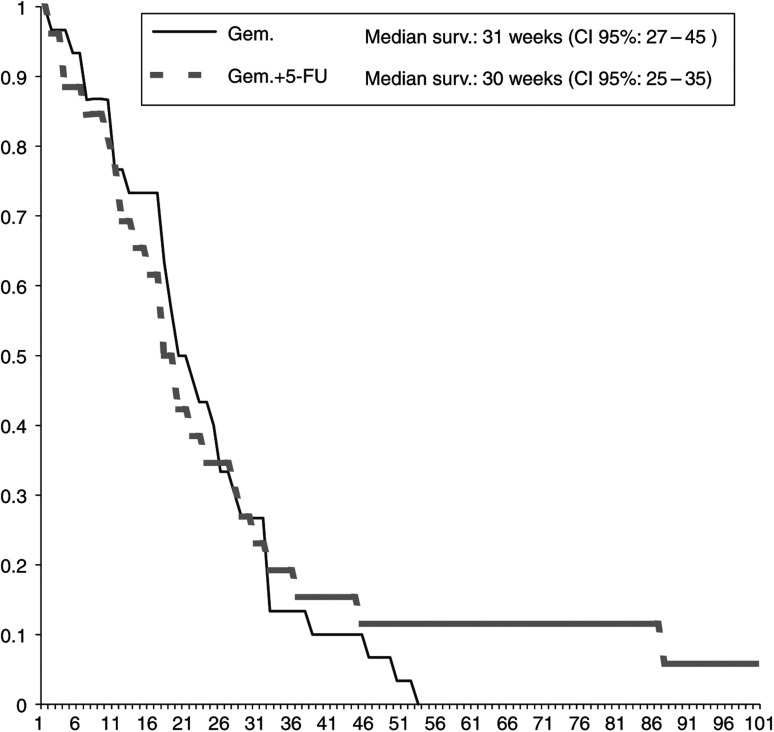
Survival curves estimated by the Kaplan–Meier method: *Y*-axis, probability of survival; *X*-axis, weeks.

In total, 91 patients were evaluable for response: 48 (98%) and 43 (96%) patients in arms A and B, respectively. Two RR were obtained in the first stage of study according to statistical considerations. The OR rate was 9.9%, with a total of nine partial responses (Table 3). There were no complete responses observed in this study. In the patients treated with GEM alone, the overall RR was 8% (95% confidence interval, 0.5–16%), while in the combination arm, it was 11% (95% confidence interval, 2–22%). The median duration of response was 34 weeks for GEM and 26 weeks for CI 5-FU+GEM. The median PFS was 14 weeks (range: 2–65 weeks) in the GEM arm alone and 18 weeks (range: 4–51 weeks) in the combination arm. Treatment with CI 5-FU plus GEM obtained a median OS of 31 weeks and 30 weeks in GEM alone (Figure 1). In all, 18% (arm A) and 20% (arm B) of patients survived over 12 months. Patients were evaluated before each cycle with QoL by SCI questionnaire. The analysis of the first cycles of treatment showed no significant increase of mean disturbed days (6.3 *vs* 4.8) and mean of days that patients would like to cancel during the treatment (4.2 *vs* 3.0) in the GEM alone and recombination arm. In the second cycle, the mean of disturbed days was 4.2 *vs* 3.0 and cancelled days 0.2 *vs* 1.2, respectively.

## DISCUSSION

Despite an increasing understanding of biology, diagnostic technology and introduction of new drugs, the OS of patients with APC remains poor. Until recently, 5-FU was considered one of the standard agents in the treatment of APC and improved therapeutic index was obtained using CI schedule.

Single-agent GEM is the currently recommended first-line treatment for APC with an RR of 10% (range 5.4–14.3%), median survival of 4.5 months and 1-year survival of 15–18%.

In a phase III trial, which compared 5-FU bolus with GEM, survival (18 *vs* 2% at 1 year) and clinical benefit response (24 and 4.4%) were improved in the GEM arm. In a compassionate trial of 3023 patients treated with GEM, Storniolo *et al* (1999) reported a median survival of 5.1 months in chemo-naïve and 3.9 in previously treated patients.

*In vitro* studies, in HT-29 colon cancer cells, revealed synergy when 5-FU was administered prior to GEM but not with concurrent administration (Shulz *et al*, 1998; Madajewicz *et al*, 2000). GEM depletes cellular deoxyuridine monophosphate (dUMP) pools, thereby decreasing competition with 5FdUMP at the target enzyme thymidylate synthase (TS). GEM may also inhibit TS. Finally, 5-FU metabolites may inhibit deoxycytidine monophosphate deaminase, an enzyme responsible for the inactivation of GEM monophosphate (Heinemann, 2002; GEM-based combination treatment of pancreatic cancer). Preclinical data do not evaluate the different methods of 5-FU administration such as protracted CI therapy *vs* bolus therapy. 5-FU bolus have reported disappointing results with a median survival of less than 5 months. More prolonged infusion schedules are associated with a median OS time of 6–8 months

The combination of GEM and 5-FU have been used in a great variety of schedules: 5-FU bolus, CI, standard-dose 5-FU or protracted (24–48 h) infusion of high-dose 5-FU given either at weekly or biweekly intervals (Castellano *et al*, 2000; Louvet *et al*, 2001; Maurel *et al*, 2001).

Six studies, including 164 patients with APC, have evaluated CI 5-FU (200–250 mg m^−2^ day^−1^) in combination with GEM in APC (Hidalgo *et al*, 1999; Anchisi *et al*, 2000; Shulman *et al*, 2000; Alabiso *et al*, 2001; Rauch *et al*, 2001; Reni *et al*, 2001). Response rate was between 13 and 51%, and OS between 5.3 and 10.3 months. Clinical benefit was only determined in three trials with an RR of 31, 45 and 78% (Hidalgo *et al*, 1999; Alabiso *et al*, 2001; Reni *et al*, 2001). Kurtz *et al* (2000), using GEM plus CI 5-FU, reported an RR of 10 and 42% of stable disease (SD) with a median survival of 4 months. GEM plus CI 5-FU was well tolerated and major toxicities were principally mucositis, nausea/vomiting and leukopenia. The toxicities of CI 5-FU and GEM do not overlap, they can be used in combination at full doses, as demonstrated by Hidalgo *et al* (1999). Recent data from studies comparing GEM with or without 5-FU suggest that 5-FU bolus administration may be of little or no benefit.

Berlin *et al* in a randomised trial of 327 patients used GEM alone weekly for 3 weeks out of every 4 weeks or GEM followed by 5-FU weekly on the same schedule (Berlin *et al*, 2002). The combination arm did not improve the median OS (6.7 *vs* 5.4 months) compared with GEM alone. The RR were in the range of 5.6 and 6.9% and PFS 2.2 months compared with 3.4 months for the combination. This study did not demonstrate a synergistic activity of this combination with respect to GEM alone. This trial demonstrated that GEM plus 5-FU is a tolerable regimen. Although toxicities were more common on the GEM plus 5-FU arm, there was no significant difference between the two arms. The most common grade 3 and 4 side effects were leukopenia, thrombocytopenia and diarrhoea. Other trials using 5-FU bolus plus GEM reported a median response of 14% (range 3.7–20%) and a median survival of 6.4 months (range 4.4–7.5 months) (Gutzler *et al*, 1999; Castellano *et al*, 2000).

Our research group investigated a combination regimen of GEM plus CI 5-FU *vs* GEM alone in a randomised phase II trial in APC. The results do not suggest a better activity of combination over GEM alone. The overall RR for all patients treated was 8% in GEM alone and 11% in the combination arm. The evaluation of tumour areas in pancreatic cancer is difficult, even with newer imaging techniques, because of a vigorous desmoplastic reaction, including inflammation and fibrosis within and around the tumour. In the locally advanced pancreatic cancer, we used CT scan to parameter the lesion with two perpendicular diameters, possibly excluding desmoplastic reaction even if there were possible difficulties in accuracy. In this trial, we did not obtain any objective response in the primary tumour. Finally, median TTP and OS are similar in both arms.

Historical comparisons between clinical trial are hazardous because there is considerable variability between study population, schedule and methodology of response evaluation, although with these considerations in mind the median survival for GEM alone, on GOIRC trial, was better than the principal studies with GEM alone (7 *vs* 5.7 months). The RR and OS in trials with GEM plus 5-FU bolus ranged from 3.7 to 20% and 5–10 months *vs* 13–25% and 4–7 months with GEM plus CI 5-FU, respectively.

The incidence of gastrointestinal grade 3 and 4 toxicities was low in both arms. Haematological toxicity was principally grades 1 and 2; only one patient (2%) developed severe thrombocytopenia. Clinical benefit response, as introduced by Burris, was not measured prospectively in this study. In this trial, patients with disease confined to the pancreas alone (33% in the combination arm and 27% in GEM alone) did not receive radiotherapy. Although the more recent studies suggest a reasonable survival time with CT+RT, the results are not convincingly better than chemotherapy alone. Thus, it follows that it is not clear whether CT+RT confers any survival advantage when compared with CT alone (Neoptolemos *et al*, 2003). The patients were evaluated for QoL by SCI questionnaire and there were no statistical differences between the two arms, although only a small number of patients received an evaluation for QoL after second cycles due to progression of disease.

In conclusion, treatment of APC remains a challenge. This trial demonstrated that the addition of CI 5-FU to GEM does not show sufficient activity in order to be further tested in phase III trials. Other trials with several different schedules or modulations of 5-FU do not improve the outcome of pancreatic cancer. A future goal could be represented by the introduction of targeted therapies to improve disease control without significant added toxicity.

## Figures and Tables

**Table 1:** Patient characteristics

	**GEM**		**GEM+CI 5-FU**	
**Characteristics**	**No.**	**(%)**	**No.**	**(%)**
Enrolled	49		45	
Evaluable	48	98	43	96
				
*Median age (years)* (range)	64 (34–75)		62 (44–75)	
Gender				
Male	23	48	27	63
Female	25	52	16	37
				
*PS (Karnofsky)*				
⩾80	33	69	29	67
<80	15	31	14	33
				
*Previous surgery*				
Yes	21	44	17	40
No	27	56	26	60
				
*Site of disease*				
Pancreas alone	13	27	14	33
Pancreas+liver	28	58	20	47
Pancreas+nodes	6	13	6	14
Others	1	1	3	7
				
*Disease at presentation*				
Locally advanced	13	27	14	33
Metastatic disease	35	73	29	67

GEM=gemcitabine; CI=continuous infusion; 5-FU=5-fluorouracil; PS=performance status.

**Table 2:** Grade of adverse effects by treatment group

	**GEM, *N*=49**			**GEM+CI 5-FU, *N*=41**		
	**Grade (WHO)**					
**Type of toxicity**	**1+2**	**3**	**4**	**1+2**	**3**	**4**
*Hematological*						
WBC	28 (57%)	1 (2%)	—	17 (41%)	1 (2%)	—
Haemoglobin	24 (49%)	3 (6%)	—	14 (34%)	3 (7%)	—
Platelets	7 (14%)	—	—	8 (20%)	—	1 (2%)
						
*Nonhaematological*						
Nausea/vomiting	21 (43%)	—	—	13 (32%)	1 (2%)	—
Mucositis	7 (14%)	—	—	12 (29%)	2 (5%)	—
Diarrhoea	2 (4%)	—	—	8 (20%)	—	—
Asthenia	20 (41%)	1 (2%)	—	13 (32%)	1 (2%)	—
Fever	9 (18%)	1 (2%)	—	7 (17%)	2 (5%)	—

GEM=gemcitabine; CI=continuous infusion; 5-FU=5-fluorouracil.

**Table 3:** Objective response rate, duration of response, PFS and survival data

**Variable**	**GEM, *n* (%)**	**GEM+CI 5-FU, *n* (%)**
Evaluable patients	48	43
CR	—	—
PR	4 (8%)	5 (11%)
(95% confidence interval)	(0.5–16%)	(2–22%)
SD	14 (29%)	12 (28%)
PR+SD	18 (37%)	17 (39%)
PD	30 (63%)	26 (61%)
Median duration response (range)	34 weeks (25–101)	26 weeks (16–46)
Median PFS (range)	14 weeks (2–65)	18 weeks (4–51)
Median OS (range)	31 weeks (1–101)	30 weeks (1–101)

PFS=progression free survival; CR=complete response; PR=partial response; SD=stable disease; PD=progression disease.
